# Investigating the effect of butanol isomers on combustion and emissions in port injection dual fuel diesel engines: an experimental study

**DOI:** 10.1371/journal.pone.0326197

**Published:** 2025-06-18

**Authors:** Mehmet Ferruh Kılınç, Gökhan Öztürk, Müjdat Fırat

**Affiliations:** 1 Support Services Directorate, Elazığ Municipality, Elazığ, Türkiye; 2 Technical Vocational School, Batman University, Batman, Türkiye; 3 Department of Automotive Engineering, Faculty of Technology, Fırat University, Elazığ, Türkiye; Tecnológico de Monterrey, MEXICO

## Abstract

The present study examines the effects of substituting alternative fuels for diesel fuel and employing a dual fuel approach on diesel engine combustion characteristics and emissions. Various butanol isomers, namely ıso-butanol, n-butanol, tert-butanol, and sec-butanol, were chosen as novelty alternative fuels. In dual fuel combustion strategy, diesel fuel was injected directly into the cylinder, while butanol isomers as a secondary fuel were introduced into the cylinder at the beginning of the intake period using a port injection technique. The tests were repeated for 15%, 30%, and 45% premixing ratios (Rp) of butanol isomers. This study presents detailed combustion parameters and pollutant emission findings produced in diesel engines employing a dual fuel strategy with butanol isomers. In general, an increase within in cylinder pressure and heat release rate was observed. Especially at a premixing ratio of 45%, an increase of 50% within heat release rate was observed. Use of all butanol isomers increased the ignition delay and shortened combustion duration. Brake thermal efficiency remained at acceptable levels, and ringing intensity was below the knock limit. In addition to an increase in CO and HC emissions, NOX emissions were also up at other premixing ratios but declined at 15%. High levels of decreased smoke opacity were recorded. Especially at a premixing ratio of 45% iso-butanol, a decrease up to 90% is remarkable. In conclusion, the combustion characteristics and pollutant emission results obtained from the experimental engine are discussed in detail according to the operating parameters. The obtained findings provide important information about the performance and emission profiles of alternative fuels and dual fuel systems and provide guidance for future research.

## 1. Introduction

Global population growth is driving up industrial construction and energy use [1]. This increasing demand is rapidly reducing fossil fuel reserves and accelerating research on alternative fuels [2]. Today, energy needs are largely met by fossil fuels, and this situation causes environmental problems by increasing harmful emissions into the atmosphere. Due to these negative effects, researchers have been searching for renewable, economical, and environmentally friendly energy sources [3–5]. Several methods can be used to obtain renewable energy sources. Nevertheless common disadvantage of these sources is that their power output cannot be predicted due to natural fluctuations, and therefore their production cannot be programmed [6].

Because of their high oxygen concentration, alcohols are favored as an alternative fuel when combined with diesel fuel. Short-chain alcohols (having three carbon atoms or less) have the advantages of lowering emission levels and improving combustion performance, but their low cetane numbers and high vaporization latent heat cause stability problems in diesel engines [7,8]. Long-chain alcohols (having a minimum of four carbon atoms) have the advantage of high cetane numbers and energy content when used as additives in diesel engine fuels. In addition, they offer benefits such as abrupt combustion phase realization and improvement of diffusion combustion phase due to their low oxygen content [9]. Among the long-chain alcohols, butanol is one of the alcohols that attracts the most attention as alternative fuel within diesel engines [10]. In the literature, separate studies have been carried out for four different butanol isomers. These isomers are iso-butanol, tert-butanol, n-butanol and sec-butanol, although their thermal values and chemical formulas are the same, their molecular structures different [11]. In addition, studies in which all isomers are examined together are rarely seen in the literature. Pan et. al. investigated combustion and emission effects of new fuel mixtures obtained by mixing butanol isomers ıso-butanol, n-butanol, tert-butanol and sec-butanol with pure diesel high ratios within common rail diesel engine. The results presented that the highest heat release rate at high loads was obtained for the diesel/secbutanol blend. The sequence of ignition delay for blends containing butanol was established as follows: ıso-butanol, sec-butanol, tert-butanol and n-butanol arranged from the most prolonged to the shortest delay period. The tert-butanol mixture exhibited superior brake thermal efficiency in contrast to diesel at elevated loads. Furthermore, the employment of different butanol isomers led to a decrease in emission levels. [11]. Han and Somers examined the properties of combustion of ıso-butanol, tert-butanol, and n-butanol isomers when mixed with diesel at different proportions. The investigation was performed utilizing a constant volume chamber within parameters resembling those of an internal combustion engine. The outcome indicated that tert-butanol/diesel mixture exhibited the quickest ignition and the most significant initial heat release. Nonetheless, the ignition delay lengthens with higher butanol concentrations and reduced ambient temperature. Additionally, the tert-butanol/diesel combination is well-suited for operating under light loads and during idle periods, offering the possibility of mitigating soot formation [12]. He et al. explored an investigation on particulate matter emissions resulting from the use of different butanol isomers within compression ignition engines. Four distinct butanol isomers, namely iso – butanol, n-butanol, tert-butanol and sec-butanol were blended into diesel fuel at concentration of 25%. The research revealed that with a rise in engine load, the particulate matter emissions of all fuel blends exhibited an initial decrease followed by a subsequent rise. The findings indicated that at lower loads, butanol isomers generated higher levels of particulate matter along with larger particles. Furthermore, it was documented that the implementation of butanol isomers led to a reduction within both soot emissions and NO_X_ emissions by 86% during low-temperature combustion [13].

The use of butanol in internal combustion engines is widely discussed in the literature [14–18]. This interest stems from butanol’s environmentally friendly properties and the fact that it is seen as a more sustainable alternative to fossil fuels [19,20]. The evaluation of butanol as a potential biofuel in both diesel and petrol engines is of great importance for energy security and environmental sustainability [21–23].

In addition to research on renewable energy sources, studies are also carried out to improve the performance of existing systems and to develop various combustion strategies. In the realm of literature, various combustion strategy approaches are employed, including premixed charge compression ıgnition (PPCI), dual fuel sequential combustion (DFSC), direct dual fuel stratification (DDFS), reactivity-controlled compression ignition (RCCI), intelligent charge compression ignition (ICCI) and homogeneous charge compression ignition (HCCI) each utilized with distinct application methodologies [24]. Especially the abandonment of diesel engines due to emission problems has increased the importance of research on these engines. In addition to combustion strategies on diesel engines, emission values are tried to be reduced by using dual fuels. Various alcohols, such as ammonia, hydrogen, and ethanol, which are seen as more environmentally friendly fuels, are used as the second fuel [25].

Cai and et. al. conducted experiments with two different applications of the n-butanol/diesel dual fuel combustion process: dual direct injection (DI^2^) and RCCI strategies. They reported that the DI^2^ strategy kept NO_X_ emissions at low levels while reducing soot emissions to almost zero and maintaining fuel consumption at low levels. They did discover, however, that because of the greater in-cylinder temperature and local equivalent ratio in DI^2^ compared to RCCI, NO_X_ emissions were higher. They also stated that the DI^2^ strategy has ıncreased combustion efficiency and decrease HC and CO emissions are observed at low levels [26]. Aravind et al. investigated the effects of a combination of hydrogen-enriched algal Spirogyra biodiesel, di-tert-butyl peroxide, and algal-derived carbon nanoparticles on the performance of a single-cylinder dual-fuel diesel engine. They observed that increasing the hydrogen flow rate improved combustion efficiency and raised exhaust gas temperature. The addition of hydrogen also increased in-cylinder pressure and heat release rate, while reducing ignition delay and combustion duration. Replacing diesel fuel with this blend significantly decreased CO, HC, and smoke emissions but caused an increase in NOx emissions. Furthermore, a decrease in volumetric efficiency was noted at higher hydrogen fractions [27]. Zhang et al. investigated the effects of n-butanol and coal liquid fuel dual fuel mode on the combustion and emission characteristics of a CI engine using a dual fuel engine with flexible combustion boundary conditions. The results of the research revealed that the ignition delay of coal-to-liquid fuel is shorter and helps reduce emissions. It was also found that n-butanol pre-sprayed into the intake system increased the premixed combustion rate and reduced particulate emissions. It was observed that the EGR system improved the effects of n-butanol and reduced NOx emissions by 49.5% and particulate matter emissions by 40.9%. It was also stated that pre-spray n-butanol mitigated the negative effects of EGR on HC emissions [28]. Le et al. carried out research on the effect of a diesel/methanol dual-fuel direct injection engine on combustion and emissions. The evaporation of methanol was found to enhance the engine’s efficiency, with emissions being influenced by adjustments in injection parameters. Throughout the experimental investigations, the augmentation of methanol ratio demonstrated a rise in engine efficiency and NO_X_ emissions, alongside a decrease within CO and soot emissions. [29]. Zhang and co-workers carried out a comparative analysis by detailing the three combustion modes of a diesel and alcohol dual-fuel engine: conventional dual-fuel (CDF), RCCI, and high-pressure direct injection (HPDI). They observed that the CDF mode is more favorable under high load conditions and NOx and PM emissions are lower compared to a pure diesel engine. In RCCI mode, the diesel and alcohol dual-fuel engine provides higher thermal efficiency while reducing NOx and soot emissions; however, combustion stability decreases and HC and CO emissions increase. In HPDI mode, the diesel and alcohol dual-fueled engine is less prone to knocking, and higher compression ratios and alcohol substitution ratios can be used in this mode [30].

The objective of this study is to thoroughly examine utilization of butanol isomers as a alternative fuel within diesel engines. This research aims to evaluate the effects of butanol isomers on engine performance and exhaust emissions, with particular emphasis on their potential to reduce pollutant formation. Furthermore, the study emphasizes the practical applicability of butanol isomers by examining their integration into conventional diesel engine systems, thereby bridging the gap between laboratory research and real-world engine operation.

In addition, the study seeks to explore the implementation of butanol isomers as secondary fuels through a dual-fuel approach in compression ignition engines, with the intent of providing a novel contribution to the existing body of knowledge. This approach is expected to inform future fuel design and engine calibration strategies aimed at enhancing combustion efficiency and environmental compliance. Contrary to the majority of studies in the literature, the present work employs port fuel injection for the introduction of the secondary fuel, rather than the more commonly used blending method. This methodological deviation represents a significant point of distinction. The use of port injection not only introduces an innovative experimental technique, but also offers a technically viable and industrially scalable alternative for the incorporation of alcohol-based fuels into existing engine architectures. As a result, taking into account the favorable thermophysical properties of butanol and its environmental advantages cited in the literature, it is anticipated that its use as a secondary fuel will substantially reduce soot emissions in compression ignition engines. This potential for soot mitigation aligns with current regulatory demands and positions butanol isomers as promising candidates for sustainable engine technologies.

## 2. Materials and methods

In this study, experimental research was conducted to achieve an efficient and clean combustion process through low-temperature combustion in diesel engines. This was accomplished using a dual fuel system to minimize the environmental damage caused by pollutants generated in the automotive industry.

In the current investigation, a compression ignition engine with a single cylinder was utilized. The information pertaining to the engine can be found in Table 1. Throughout the experimental procedures, an electric dynamometer was employed to administer required load to engine. The single-cylinder engine, coupled with this dynamometer, was powered by a common rail fuel injection system.

**Table 1:** Detailed technical parameters of the test engine.

Engine type	Common rail direct injection
**Number Of Cylinder**	1
**Compression Ratio**	18;1;1
**Cylinder Diameter x Strok (mm)**	86x70
**Cylinder Volume (L)**	0,406
**Power output @ 3000 rpm (HP)**	10
**Max. Torque@2400 rpm (Nm)**	25.7
**Injection pressure(Bar)**	300

**Fig 1:**
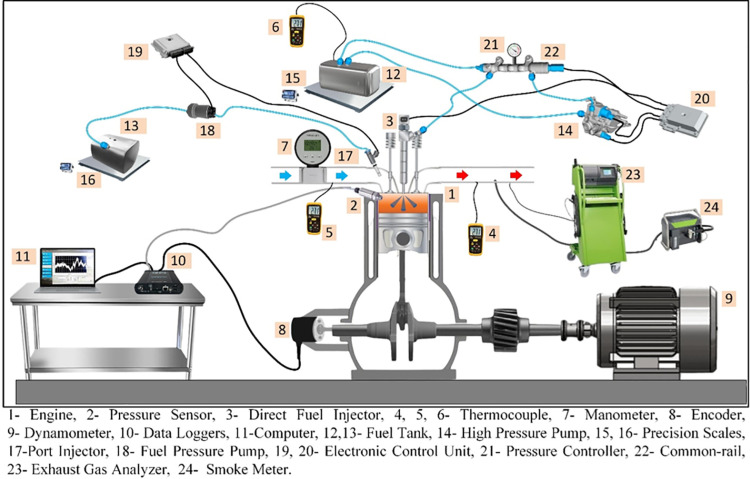
Diagram illustrating the experimental setup.

To measure exhaust emission and smoke opacity, BEA 350 and Bosch RT430 devices were used. A general view of the experimental set is shown in Fig 1. Combustion analyses were performed using Febris software. During crankshaft rotation, a Kuebler encoder is used to determine the position of the crankshaft. To measure the in-cylinder pressure, the Optrand D33288-GPA model pressure sensor is mounted on the cylinder head. The pressure data obtained from this sensor was recorded to a computer via an NI USB-6351 data acquisition card. In the combustion analyses, the in-cylinder pressure data were evaluated with an average of 200 cycles. The secondary fuel system consists of a port-type injector placed in the intake port and a fuel pump operating at 5 bar pressure. This system allows the injector to inject fuel into the cylinder at specified engine speed and time.

In the conducted experiments, diesel fuel served as the primary fuel, while butanol isomers were chosen as secondary fuels. The specific isomers used include n-butanol, sec-butanol, iso-butanol, and tert-butanol. Table 2 provides a comprehensive overview of the chemical and physical characteristics of these fuels.

**Table 2:** Chemical and physical attributes of diesel fuel and butanol isomers [11,31,32].

Parameters	Diesel	n-butanol	sec-butanol	iso-butanol	tert-butanol
Molecular Formula	C_12_-C_25_	C_4_H_9_OH	C_4_H_9_OH	C_4_H_9_OH	C_4_H_9_OH
Molecular weight (kg/kmol)	208.20	74.12	74.12	74.12	74.12
Density (kg/m^3^)	842.0	809.8	806.3	801.8	788.7
Boiling temperature (°C)	180-385	117.4	99.5	108.0	82.4
Lower heating value (MJ/kg)	42.8	33.19	32.74	33.11	29.79
Standard enthalpy of vaporization (kJ/kg)	270	626	671	684	511
Self-ignition temperature (°C)	200-250	343	406.1	415.6	477.8
Carbon content (%)	86.74	64.86	64.86	64.86	64.86
Hydrogen content (%)	13.26	13.51	13.51	13.51	13.51
Oxygen content (%)	–	21.6	21.6	21.6	21.6
Octane number	–	96	101	113	105
Cetane number	51-59	12.0	8.5	8.5	5.6

Experiments in this study were conducted at 50% engine load to evaluate the diesel engine’s performance under medium-load conditions. The engine load percentage was determined using the maximum torque value of 25.7 Nm, as shown in Table 1. Testing was performed with a maximum engine speed of 2400 rpm and with fixed injection timings. The operational parameters are detailed in Table 3.

**Table 3:** Experimental Study Parameters.

Parameters	Reference Experiments	Dual Fuel Experiments
**Engine load(%)**	50	50
**Engine speed(rpm)**	2400	2400
**Diesel injection pressure(bar)**	300	300
**Secondary fuel injection pressure (bar)**	–	5
**Diesel injection time (°CA)**	21	21
**Mixing ratio % (of total energy content)**	–	15, 30 and 45
**Second fuel injection timing (°CA)**	–	15
**Main fuel**	Diesel	Diesel
**Secondary fuel**	–	n-butanol sec-butanol iso-butanol tert-butanol

The mixing ratio of the fuels in the experiments was determined using the energy per cycle method. This approach calculates the mixing ratio based on the total energy provided. Given the potential inaccuracies of volumetric methods due to the variable thermophysical properties of different fuels, the energy supplied to the engine per cycle was maintained constant to ensure accuracy. Consequently, the mixture ratio was derived from this consistent energy input. The calculation procedure is outlined in Equation (1) below.


$$\text{Rp= }\frac{{\text{M}}_{\text{Butanol}}\;\text{x }{\;\text{Hu}}_{\text{Butanol}}}{{\text{M}}_{\text{Butanol }}\text{x }{\text{Hu}}_{\text{Butanol}}\text{ + }{\text{M}}_{\text{Diesel }}\text{x }{\text{Hu}}_{\text{Diesel}}\;}$$ (1)

In this equation, Rp is the mixing ratio, and M and Hu are the mass and lower heating values of the fuels, respectively [33].

According to equation (2), combustion analysis software calculates heat release rate by using the first law of thermodynamics and in-cylinder pressure data;


$$\begin{array}{c} \frac{\text{dQ}}{\text{d}\theta }\text{=}\frac{\Upsilon }{\Upsilon \text{-1}}\text{p}\frac{\text{dV}}{\text{d}\theta }\text{+}\frac{\text{1}}{\Upsilon \text{-1}}\text{V}\frac{\text{dp}}{\text{d}\theta } \end{array}$$ (2)

where, γ specific heat ratio, θ is the crank angle, P is the cylinder pressure, and V is the cylinder volume.

Within the scope of the study, ringing intensity (RI), which is important data related to engine performance, was calculated. Ringing intensity was calculated using equation (3).


$$\begin{array}{c} RI\approx \frac{{\left(\beta .{\left(\frac{dP}{dt}\right)}_{\max}\right)}^{2}.\sqrt{\Upsilon .R{.T}_{\max}}}{2.\Upsilon .P_{\max}} \end{array}$$ (3)

RI stands for Ringing Intensity, with R reflecting the ideal gas constant, γ defining the specific heat rate, P_max_ indicating the maximum cylinder pressure and T_max_ representing the maximum in-cylinder temperature$.{\left(\frac{dP}{dt}\right)}_{\max}$ signifies the maximum pressure rise rate, while β is an empirically determined scaling coefficient of pressure oscillations [34].

Brake thermal efficiency ($\eta_{\text{t}}$) was computed according to equation (4).


$$\eta_{\text{t}}=\frac{W_{net}}{{(\dot{\text{m}}}_{diesel}\;x{\;Q}_{LHV\;diesel})+{(\dot{\text{m}}}_{\text{Butanol}}\;x{\;Q}_{LHV\;\text{Butanol}})}$$ (4)

$W_{net}\;$ represents the net work done, ${\dot{\text{m}}}_{diesel}$denotes the mass flow rate of diesel, ${\dot{\text{m}}}_{\text{Butanol}}$ indicates the mass flow rate of butanol, ${\;Q}_{LHV\;diesel}$ is the lower heating value of diesel, and ${\;Q}_{LHV\;\text{Butanol}}$ refers to the lower heating value of butanol [35].

Uncertainty calculations are made through equation 4.


$$w_{R}={\left[{\left(\frac{\partial R}{\partial x_{1}}w_{1}\right)}^{2}+{\left(\frac{\partial R}{\partial x_{2}}w_{2}\right)}^{2}+\ldots +{\left(\frac{\partial R}{\partial x_{n}}w_{n}\right)}^{2}\right]}^{\frac{1}{2}}$$ (5)

The uncertainty value, denoted as w_R_, reflects the variability in the results and depends on the independent variables x_1_, x_2_,…, x_n_ and their associated uncertainties w_1_, w_2_, …, w_n_. This relationship is outlined in reference [36].

Using equation 4, the uncertainty was calculated as follows

$w_{R}=\sqrt{(0,0119{)}^{2}+(0,00989{)}^{2}+(0,0033{)}^{2}+(0,00014{)}^{2}+(0,0008{)}^{2}+(0,0128{)}^{2}}\;$ = 0,0386 was found. The overall uncertainty for brake-thermal efficiency, fuel consumption, and pollutant emissions, derived from experimental measurements, is calculated to be 3.86%.

## 3. Result and discussions

In this section, the results of combustion and emission formation under constant engine load and speed for four different butanol isomers and their three different premixing ratios are presented graphically. The presented graphs are discussed based on the literature.

**Fig 2:**
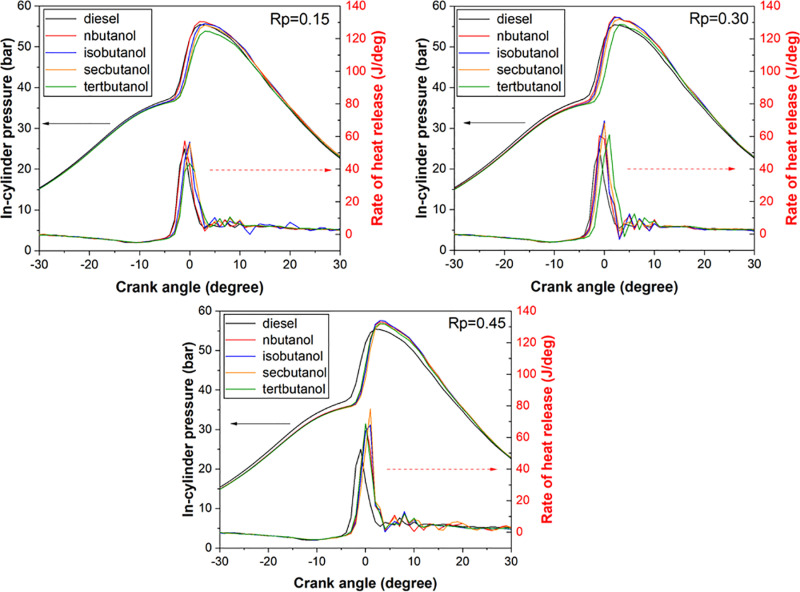
Changes in in-cylinder pressure and heat release rate with respect to different butanol isomers and their premixing ratios.

Fig 2 illustrates the relationship between in-cylinder pressure and heat release rate concerning crank angle across three distinct charts based on various butanol isomers and premixing ratios. It is generally noted that the utilization of butanol isomers as fuel leads to a rise in both in-cylinder pressure and heat release rate across all premixing ratios. This escalation can reach levels of up to 4% for in-cylinder pressure and 50% for heat release rate, depending on the increasing premixing ratios. The interpretation of these graphs is deemed accurate, considering that diesel fuel undergoes direct injection into the cylinder while butanol isomers are introduced via the port injection method during intake. The study attributes these increments to the inherent spontaneous ignition properties of butanol isomers, where their high octane numbers and autoignition temperatures significantly influence the outcomes. Notably, among the butanol isomers, the lower autoignition temperature of n-butanol results in premature ignition and elevated in-cylinder pressure across all blending scenarios. Moreover, the extension of the combustion initiation period positively impacts the fuel-air mixture, thereby supporting the augmentation of the heat release rate. A study conducted by He and colleagues documented a noticeable upsurge in in-cylinder pressure and heat release rate following the incorporation of butanol isomers in diesel engines [13].

**Fig 3:**
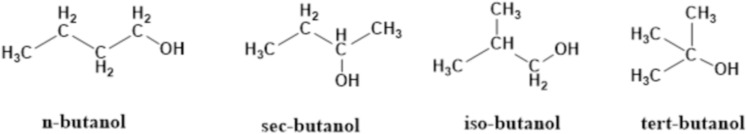
Open chain representations of butanol isomers.

Fig 3 presents the open-chain structures of butanol isomers. Among the butanol isomers, n-butanol has the least branched structure, whereas tert-butanol exhibits the most compact and highly branched structure. Among the butanol isomers, n-butanol produced the highest in-cylinder pressure, while tert-butanol resulted in the lowest. This can be explained by the differences in their cetane numbers, which are influenced by molecular structure. As branching in hydrocarbons increases, the cetane number tends to decrease [37]. The higher cetane number of the straight-chain n-butanol compared to its more branched isomers contributes to more favorable combustion characteristics, resulting in increased in-cylinder pressure.

**Fig 4:**
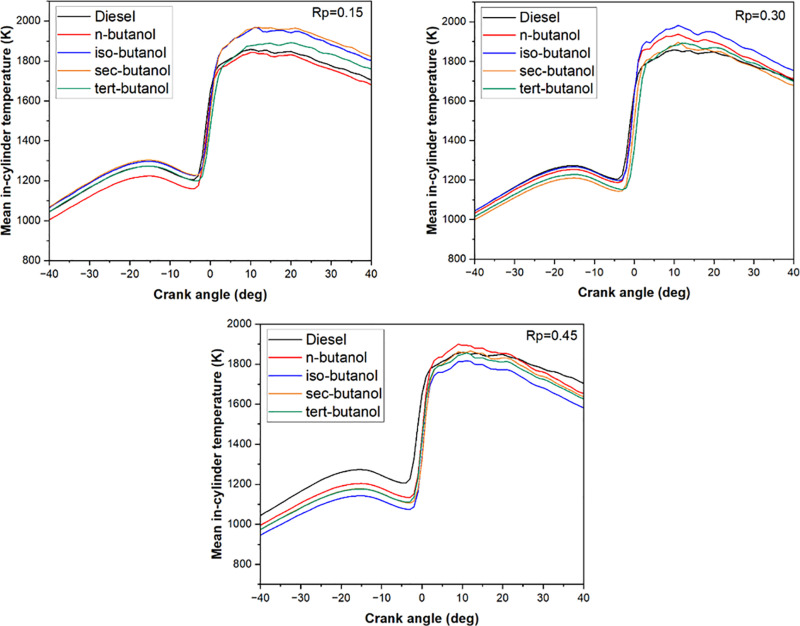
Variation of average gas temperature depending on butanol isomers and premixing ratio.

Fig 4 shows the variation of in-cylinder average gas temperatures for four different butanol isomers and three different premixing ratios. In this study, the engine load was kept constant at 50% of the maximum engine torque. Therefore, tests were carried out under medium-load conditions. The increase in maximum combustion temperatures is an expected result in dual fuel studies using port injection techniques and is frequently seen in the literature [38–40]. The port injection of butanol isomers resulted in an increase in in-cylinder temperature compared to the reference fuel. This improvement in thermal performance is primarily attributed to the oxygen content in butanol’s molecular structure, which promotes more efficient combustion. Additionally, structural differences among the isomers influence their physicochemical properties—such as cetane number, volatility, and heating value—that affect the combustion process [41]. For instance, the straight-chain structure of n-butanol is associated with a higher cetane number and more favorable combustion characteristics, leading to higher in-cylinder temperatures. In contrast, branched isomers like tert-butanol exhibited slightly lower thermal performance. Due to its higher volatility compared to diesel fuel, butanol formed a more homogeneous air–fuel mixture, which contributed to an increase in in-cylinder temperature. Saba et al. reported that heterogeneous mixtures tend to deteriorate combustion quality and negatively affect emission characteristics [42]. When the graphs are analyzed, it is seen that as the mixture ratio increases, in-cylinder temperatures before the top dead point decrease. This decrease is thought to be due to the latent heat of vaporization of butanol isomers. Low in-cylinder temperatures continue until the start and development of combustion. It was observed that the maximum temperature formation occurred for iso-butanol and sec-butanol at all premixing ratios, while the temperatures increased faster for n-butanol. It is thought that the improvement in the air-fuel mixture due to the prolongation of the ignition delay is reflected in the in-cylinder temperatures. It is also clearly seen in the graph that after the controlled combustion stage, the in-cylinder gas temperatures rapidly decreased to the level of conventional diesel combustion in the study using butanol isomers. This change was considered to be dependent on the combustion time.

**Fig 5:**
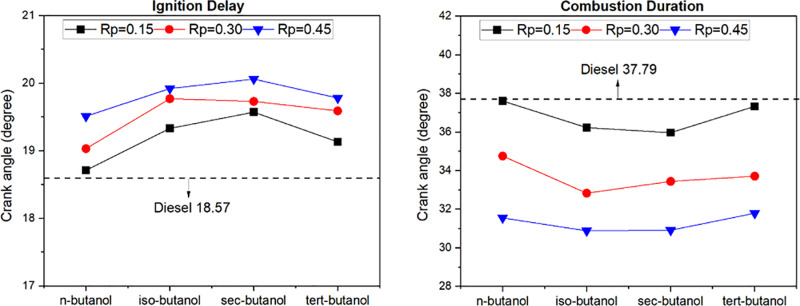
Changes in ignition delay and burning time depending on butanol isomers and mixture ratio.

Fig 5 displays the changes in ignition delay and combustion time in a butanol/diesel dual-fuel diesel engine in which butanol isomers are used as fuel by port injection technique, depending on fuel type and premixing ratio. The ignition delay, a critical parameter reflecting the initiation of combustion, is significantly influenced by various factors [43]. Research indicates that n-butanol exhibited the shortest ignition delay across all premixing ratios, followed by tert-butanol, while sec-butanol resulted in the longest ignition delay. In hydrocarbon molecules, an increase in chain length leads to a decrease in octane number [44]. Among the butanol isomers, n-butanol has the longest carbon chain, as shown in Fig 3. This trend can be attributed to the reduce octane number and auto-ignition temperature of n-butanol compared to other isomers, thereby reducing the ignition delay. Furthermore, the analysis highlights that the ignition delay is impacted by the low cetane number and latent heat of vaporization of tert-butanol. Notably, iso-butanol and sec-butanol, possessing closely aligned thermochemical properties, demonstrated similar ignition delay results. Comparatively, an extended ignition delay was observed for all premixing ratios and butanol isomers in contrast to traditional diesel operation. The combustion time correlates with changes in ignition delay, showcasing a reduction across all premixing ratios and butanol isomers. This phenomenon is attributed to enhanced air-fuel mixtures stemming from prolonged ignition delay and the impact of combustion at lower temperatures facilitated by the port injection method. Altun et al. reported similar findings, stating that the ignition delay increased with higher mixture ratios, while the combustion duration was shortened due to the evaporative effect of alcohols [45].

**Fig 6:**
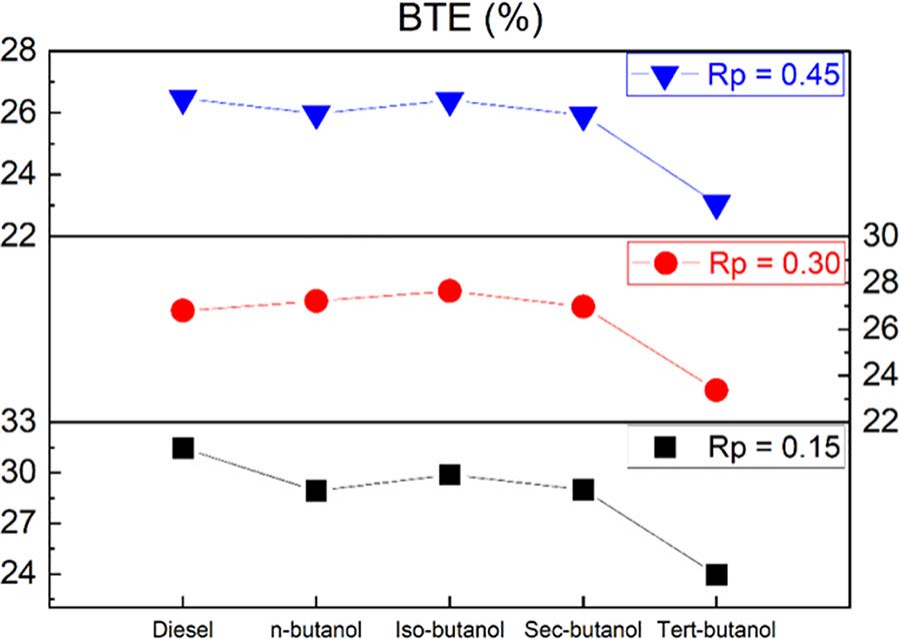
Variation of brake thermal efficiency depending on butanol isomers and premixing ratio.

Fig 6 shows the variation of brake thermal efficiency for different premixing ratios and butanol isomers. It can be seen that at a premixing ratio of 15%, the thermal efficiencies are lower compared to conventional diesel combustion. For this premixing ratio, the maximum decrease is 24% for tert-butanol, followed by n-butanol at 8%. The least change in thermal efficiency for this premixing ratio was 5% for iso-butanol. It can be deduced from the graph that the thermal efficiency tends to increase under operating conditions where the premixing ratio is 30%. For n-butanol, iso-butanol, and sec-butanol at this premixing ratio, an increase of approximately 4%, 6.5%, and 4% is observed, respectively, while a 10% decrease is observed in the case of tert-butanol. Similarly, a thermal efficiency close to conventional diesel conditions was obtained for a premixing ratio of 45%. At a 45% premixing ratio, there is a 2% increase for iso-butanol and an 11% decrease for tert-butanol. When the graph is evaluated in general, it is seen that the thermal efficiency is sustainable for the butanol isomers used under the specified engine operating conditions. Moreover, it was assessed that the thermal efficiency exhibited greater consistency with the escalation of the mixture ratio of butanol isomers. Conversely, in accordance with this investigation, iso-butanol, n-butanol, sec-butanol, and tert-butanol can be hierarchically arranged as the most suitable sequence for enhancing brake thermal efficiency. The utilization of butanol isomers with a lower heating value compared to diesel fuel is believed to result in a reduction in brake thermal efficiency. The efficiency of brake thermal is intricately linked to the calorific capacity of the fuel utilized. Essentially, with the augmentation of the energy content within the fuel, there is a corresponding elevation in the brake thermal efficiency of the engine. This association stands as one of the principal elements that impacts the functionality and energy conversion effectiveness of the engine [46].

**Fig 7:**
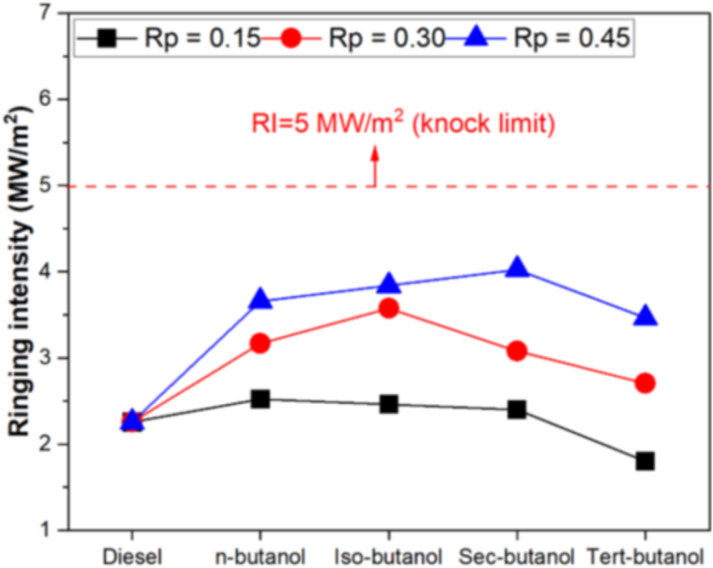
Variation of knock intensity depending on butanol isomers and premixing ratio.

Fig 7 shows the variation of knock intensity for four different butanol isomers and three different premixing ratios. The knock limit for diesel engines is emphasized in the literature as 5 MW/m^2^ [47]. According to this reference limit, it is seen that the knock intensity values obtained in all tests carried out within the scope of this study are far from the knock limit. The effect of butanol isomers on engine knock is of a nature that increases the knock tendency. However, for a 15% premixing ratio, the knock intensity is close to the conventional diesel mode. It is also noted that tert-butanol exhibits a reduced knock tendency relative to diesel. It is an important result seen from the graph that the knock tendency increases as the premixing ratio increases. When it is evaluated that the knock intensity depends on the combustion reaction rate, it is seen that air/ fuel mixture and ignition delay affect knock tendency. Especially in diesel engines, the late start of combustion increases the knock tendency.

**Fig 8:**
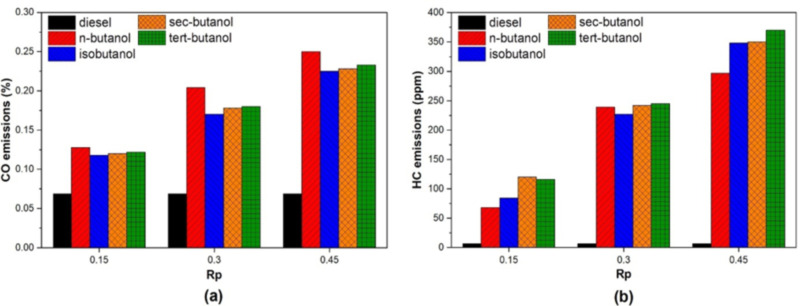
Variation of (a) CO (b) HC emissions depending on butanol isomers and premixing ratio.

Fig 8a shows the variation of CO emissions with different butanol isomers and premixing ratios. In previous studies using the port injection technique, it was seen that CO emissions increase dramatically compared to conventional diesel combustion, especially at low and medium engine loads. In the tests used in this study with 50% engine load and different isomers, it was seen that CO emissions increased significantly. It is another result that CO increases as the mixture ratio increases. Butanol isomers injected by the port injection method tend to reduce in-cylinder temperatures when the engine is used in dual fuel mode. Especially the latent heat of vaporization tends to provide this effect. This prevents complete combustion of the butanol/air mixture, especially at low and medium engine loads. Therefore, CO emissions increase as a result of flameouts and incomplete combustion, regardless of the ignition start [48]. When all the results are analyzed, although the CO emission generated by the use of iso-butanol is higher than that of diesel, the fact that it produces less emission compared to the use of other fuel blends is an expected result that is expected to contribute to the use of dual fuel mode under low and medium load conditions.

Fig 8b shows the variation of HC emissions for different butanol isomers and 15%, 30%, and 45% Rp (premixing ratios). HC emissions are a product of incomplete combustion and are affected by many factors, such as the air/fuel mixture, oxygen content, and combustion temperatures. It is known that HC emissions are higher than in diesel combustion except at high engine loads within dual fuel engine studies using port injection, competitively controlled compression ignition engines, and low temperature combustion concepts. This result has been obtained by many researchers in the literature [39,49]. In this study, it is observed that HC emissions increase dramatically with the use of butanol isomers under medium engine loads, depending on the mixture ratio. Due to the ignition delay and latent heat of vaporization of butanol isomers, it is estimated that cold conditions occur in the cylinder, and this situation significantly affects HC emissions. In addition, it was observed that flame extinction increased HC emissions due to the latent heat of vaporization [50]. In their investigation employing iso-butanol as a secondary fuel, Pan et al. observed that HC emissions increased with the blend ratio, consistent with the findings of the present study [11]. Another result obtained from the graph is that the quenching effect is lower for n-butanol and higher for tert-butanol, depending on the autoignition temperature. Despite the rise within HC emissions from the use of butanol isomers, it was found that the use of n-butanol at a mixture ratio of a mixture ratio of 15 and 45 and iso-butanol at a mixture ratio of 30 caused less HC emissions than the other mixtures. This result is considered to be important for the use of butanol isomers in dual fuel studies.

**Fig 9:**
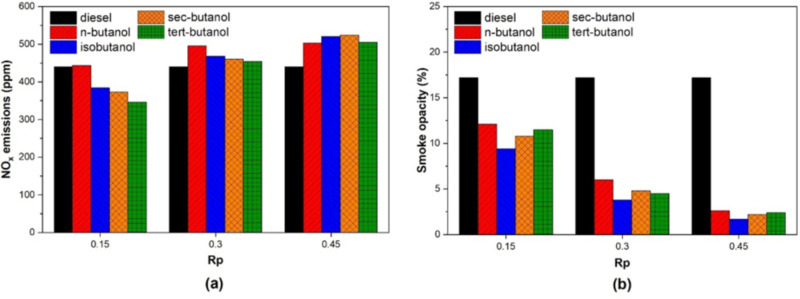
Variation of (a) NO_X_ (b) Smoke opacity emissions depending on butanol isomers and premixing ratio.

The change in NO_X_ emissions in a dual-fuel diesel engine under the conditions of using four different butanol isomers with three different premixing ratios is shown in Fig 9a. NO_X_ emissions are a type of pollutant that is strongly dependent on in-cylinder temperatures. The 50% engine load conditions applied in this study are capable of providing high in-cylinder temperatures. However, at low premixing ratios, lower NO_X_ emissions were obtained compared to conventional diesel combustion under dual-fuel operating conditions using butanol isomers due to the latent heat of vaporization effect. For a premixing ratio of 15%, the reduction in NO_X_ emissions was 13%, 16%, and 22% for iso-butanol, sec-butanol, and tert-butanol, respectively. On the other hand, an increase of 0.8% in NO_X_ emissions was observed when n-butanol was used. It is observed that NO_X_ emissions increase as the mixture ratio increases, and this increase is realized as 13% and 20% for 30% and 45% mixture ratios, respectively. While the rapid increase in in-cylinder temperatures at low premixing ratios significantly affects NO_X_ emissions, it is one of the important results obtained from this graph that NO_X_ formation at high premixing ratios, i.e., 45%, low NO_X_ levels at low premixing ratios of butanol isomers for medium engine loads, is considered promising for future studies. such as 45% is more strongly dependent on the maximum in-cylinder temperature formation. In addition, obtaining low NO_X_ levels at low premixing ratios of butanol isomers for medium engine loads is considered promising for future studies.

Fig 9b shows the effects of butanol isomers on smoke opacity depending on 15%, 30%, and 45% premixing ratios. When the graph is analyzed, it is observed that at all premixing ratios where butanol isomers are used, the smoke opacity is lower than in conventional diesel mode operation. It is clearly seen in the graph that the smoke opacity decreases as the premixing ratio increases. Altun et al. also observed comparable outcomes in their study on dual-fuel engine systems [51]. Under conditions where the premixing ratio was 15%, 30%, and 45%, the maximum reduction in smoke opacity compared to diesel fuel was 46%, 78%, and 90%, respectively. The maximum smoke opacity reduction occurred when iso-butanol was used for all premixing ratios. In the case of butanol isomers, the decrease in smoke opacity may be due to different reasons. One of these is the amount of oxygen contained in butanol isomers. This oxygen content has the effect of reducing soot emissions. Another reason is the latent heat of vaporization of butanol isomers, and it is considered that it may cause low-temperature combustion. Thus, the variation in smoke opacity between butanol isomers can be explained. These two effects have been clearly stated by researchers in previous studies [50]. In this study, the shortening of diffusion combustion due to the introduction of butanol isomers into the engine using the port injection method is seen as a factor that significantly affects the reduction of smoke opacity. The difference between butanol isomers can be explained by the chemical properties of fuels. For example, n-butanol, which has the lowest autoignition temperature among the isomers, can cause ignition in a worse air-fuel mixture and cause higher smoke opacity.

## 4. Conclusions

In this study, experimental research on dual fuel application in diesel engines was carried out. In the study, butanol was selected as an alternative fuel, and the efficiency of four different isomers of butanol was investigated. Three different premixing ratios, depending on the energy ratio of each isomer in a cycle, were experimentally investigated. The important results obtained as a result of this thesis are presented below.

- The utilization of butanol isomers as fuel in a dual-fuel engine has been noted to result in an escalation of in-cylinder pressure and heat release rate in comparison to the conventional diesel combustion process.
- Among the various butanol isomers utilized, n-butanol exhibited the shortest ignition delay, whereas sec-butanol displayed the lengthiest ignition delay circumstances. A heightened ignition delay was observed for all premixing ratios and butanol isomers when compared with the conventional diesel operation. It was noted that the combustion duration was reduced for all premixing ratios and butanol isomers.
- The effect of butanol isomers on engine knock is of the nature that increases the knock tendency. It is an important result that the knock tendency increases as the mixture ratio increases. In the tests using butanol isomers, it was evaluated that delayed combustion due to ignition delay increased the knock intensity.
- In comparison to the traditional diesel blend, a decline in brake thermal efficiency was noted at lower premixing ratios during dual fuel experiments. As the premixing ratios rose, an inclination towards matching or surpassing the brake thermal efficiency of conventional diesel was observed. The ranking for the most suitable order of employment based on brake thermal efficiency includes iso-butanol, n-butanol, sec-butanol, and tert-butanol.
- Under dual-fuel engine conditions using butanol isomers, it was observed that the average in-cylinder temperatures were lower before the start of combustion compared to conventional diesel combustion. It was observed that higher temperatures were obtained after the start of combustion with the contribution of the oxygen effect in the butanol content.
- Under dual-fuel operating conditions using butanol isomers, a significant upward trend in CO and HC emissions was observed. It is also predicted that this effect may decrease at high engine loads.
- At lower premixing ratios of butanol isomers, diminished NOx emissions were achieved in contrast to traditional diesel combustion in dual-fuel operation, attributable to the influence of latent heat of vaporization. Upon reaching a mixture ratio of 15%, NOx emissions were decreased by as much as 22%. Conversely, an escalation in NOx emissions was noted with an increase in the mixture ratio.
- Smoke opacity reduction is significantly observed to decrease with the utilization of various butanol isomers. An important discovery highlighted in the research is that employing iso-butanol at a mixing ratio of 45% resulted in a substantial 90% decrease in smoke opacity. The study’s findings rank butanol isomers, namely iso-butanol, sec-butanol, tert-butanol, and n-butanol, in order of effectiveness in reducing smoke opacity.

These results comprehensively demonstrate the effects of butanol isomers on performance and emission characteristics in dual fuel applications in diesel engines and provide important information to understand the potential advantages and limitations of alternative fuels in engine technology. Future studies can contribute to developing strategies to further improve engine performance and optimize emission control based on these findings.

## Supporting information

S1 Rp_0.30_Pressure_HRR_Temp_Dataset. (XLSX)

S2 Rp_0.30_Combustion_and_emissions_Dataset. (XLSX)
